# Newborn screening for medium-chain acyl-CoA dehydrogenase deficiency: regional experience and high incidence of carnitine deficiency

**DOI:** 10.1186/1750-1172-8-102

**Published:** 2013-07-10

**Authors:** Maria Luz Couce, Paula Sánchez-Pintos, Luisa Diogo, Elisa Leão-Teles, Esmeralda Martins, Helena Santos, Maria Amor Bueno, Carmen Delgado-Pecellín, Daisy E Castiñeiras, José A Cocho, Judit García-Villoria, Antonia Ribes, José M Fraga, Hugo Rocha

**Affiliations:** 1 Unidad de Diagnóstico y Tratamiento de Enfermedades Congénitas del Metabolismo, Departamento de Pediatría, Hospital Clínico Universitario, Universidad de Santiago, Santiago de Compostela, Spain; 2 Centro de Desenvolvimento da Criança Luís Borges, Hospital Pediátrico de Coimbra, Coimbra, Portugal; 3 Unidade Doenças Metabólicas, Hospital Pediátrico Integrado, Centro Hospitalar de S. João, EPE, Porto, Portugal; 4 Unidade de Doenças Metabólicas, Hospital de Crianças Maria Pia, Centro Hospitalar do Porto, Porto, Portugal; 5 Departamento de Pediatria, Centro Hospitalar Gaia/Espinho, Gaia, Portugal; 6 Unidad de Metabolopatías y Nutrición Infantil, Departamento de Pediatría, Hospital Universitario Virgen del Rocio, Sevilla, Spain; 7 Departamento de Bioquímica Clínica, Hospital Universitario Virgen del Rocío, Sevilla, Spain; 8 Unidad de Diagnóstico y Tratamiento de Enfermedades Congénitas del Metabolismo, Laboratorio de Metabolopatías, Hospital Clínico Universitario, Universidad de Santiago, Santiago de Compostela, Spain; 9 Sección de Errores Congénitos del Metabolismo-IBC, Servicio de Bioquímica y Genética Molecular, Hospital Clínic y Centro de Investigación Biomédica en Red de Enfermedades Raras (CIBERER), Barcelona, Spain; 10 Unidade de Rastreio Neonatal, Departamento de Genética do Instituto Nacional de Saúde Doutor Ricardo Jorge, Porto, Portugal

**Keywords:** L-carnitine, Metabolic decompensation, Mitochondrial fatty acid oxidation, Mutations, Newborn screening, Rare disease

## Abstract

**Background:**

Medium-chain acyl-CoA dehydrogenase deficiency (MCADD) is the most common inherited defect in the mitochondrial fatty acid oxidation pathway, resulting in significant morbidity and mortality in undiagnosed patients.

Newborn screening (NBS) has considerably improved MCADD outcome, but the risk of complication remains in some patients. The aim of this study was to evaluate the relationship between genotype, biochemical parameters and clinical data at diagnosis and during follow-up, in order to optimize monitoring of these patients.

**Methods:**

We carried out a multicenter study in southwest Europe, of MCADD patients detected by NBS. Evaluated NBS data included free carnitine (C0) and the acylcarnitines C8, C10, C10:1 together with C8/C2 and C8/C10 ratios, clinical presentation parameters and genotype, in 45 patients. Follow-up data included C0 levels, duration of carnitine supplementation and occurrence of metabolic crises.

**Results:**

C8/C2 ratio and C8 were the most accurate biomarkers of MCADD in NBS. We found a high number of patients homozygous for the prevalent c.985A > G mutation (75%). Moreover, in these patients C8, C8/C10 and C8/C2 were higher than in patients with other genotypes, while median value of C0 was significantly lower (23 μmol/L vs 36 μmol/L).

The average follow-up period was 43 months. To keep carnitine levels within the normal range, carnitine supplementation was required in 82% of patients, and for a longer period in patients homozygotes for the c.985A>G mutation than in patients with other genotypes (average 31 vs 18 months). Even with treatment, median C0 levels remained lower in homozygous patients than in those with other genotypes (14 μmol/L vs 22 μmol/L).

Two patients died and another three suffered a metabolic crisis, all of whom were homozygous for the c.985 A>G mutation.

**Conclusions:**

Our data show a direct association between homozygosity for c.985A>G and lower carnitine values at diagnosis, and a higher dose of carnitine supplementation for maintenance within the normal range. This study contributes to a better understanding of the relationship between genotype and phenotype in newborn patients with MCADD detected through screening which could be useful in improving follow-up strategies and clinical outcome.

## Background

Medium-chain acyl-CoA dehydrogenase deficiency (MCADD) is the most common inherited fatty acid β-oxidation (FAO) defect and is a potentially fatal disorder. FAO is a metabolic pathway of particular importance as an energy source during fasting, when glucose supply becomes limited [1]. The overall incidence of MCADD, evaluated by tandem mass spectrometry (MS/MS) newborn screening, is approximately 1:14600, which is 2- to 3-fold higher than the incidence estimated by clinical diagnosis [2]. Incidence varies widely by region, with a higher incidence in the population of northern Europe [3,4].

The MCADD phenotype ranges from asymptomatic [5] to Reye-like syndrome. Typical clinical presentation consists of a metabolic crisis, characterized by hypoketotic hypoglycemia, lethargy, coma [6], seizures or sudden death [7], and triggered by catabolic stress during fasting or illness. The disease usually manifests in the first few years [8], although first presentation in adulthood has also been described [9]. Unusual manifestations, such as neonatal ventricular tachyarrhythmias [10-12], pulmonary haemorrhage [10], and abnormal motor behavior during sleep [13] have also been described. There is a risk of neurological impairment after an acute metabolic decompensation [8,14]. The long term follow up of a Dutch cohort of clinically diagnosed cases of MCADD identified disabilities in 21% of patients [15]. The mortality rate in the first 72 hours of birth is 4%, with and additional mortality rate of 5-7% by 6 years of age in affected unscreened children [8,16].

Newborn screening (NBS) by MS/MS started in the 1990s. It has since been demonstrated to be accurate and effective [2] with a clear benefit in countries with a high percentage of Caucasians [17], contributing to a reduction in MCADD morbidity and mortality [18]. However, severe metabolic crises still occur, particularly in the early post-natal period prior to NBS [8,19,20] and before screening results are available [12,21]. Patients with fatal neonatal presentation show low residual MCAD enzyme activities (<1%) [22].

Elevations of octanoylcarnitine (C8), C8/C10 and C8/C2 ratios are the most commonly reported markers in screening for MCADD [23-26]. It is important to bear in mind that C8 values are likely to be lower when screening samples are collected 72 h after birth [27]. C8/C10 and C8/C2 ratios does not seem to be affected by time of sampling [4].

MCAD protein is encoded by the *ACADM* gene (OMIM 607008), located on chromosome 1p31. More than 80 mutations have been identified in this gene (HGMD®), most of which are missense mutations. While screening programs have greatly improved clinical outcome and contributed to current understanding of MCADD, several questions remain. Two of the most important of these are how genotype and phenotype are related [3,25,28] and the clinical relevance of novel variants of *ACADM*, as identified by newborn screening. Approximately 80% of patients diagnosed clinically are homozygous for the common c.985A>G mutation [25,29]. Patients diagnosed as a result of screening show a different mutational spectrum, with a lower proportion (30-71%) of homozygotes for the common mutation [3,20,21,23,25,27,30-35]. This diagnosis-dependent difference in frequency of the homozygous c.985A>G mutation could be due to the increased detection of milder biochemical phenotypes by newborn screening, with a relatively low risk of developing clinical disease, and associated to mutations found only in screened populations [4,31,36]. Notably, residual MCAD activity is significantly lower in patients with the common mutation (range 0-8%), compared to those with other variants (range 0-63%) [22].

Although most reports indicate that patients homozygous for the most common mutation have a poorer outcome [2,37,38], some homozygous patients who were asymptomatic until adulthood were also reported [4,8,38,39].

The main goal of the present study was to evaluate any relationships between biochemical findings at diagnosis, genotype, free carnitine (C0) levels during follow-up, and clinical outcome, in patients with MCADD detected by NBS.

## Methods

### Study population

The present study population comprised MCADD patients diagnosed by two of the Spanish regional (Galicia and western Andalusia) and one Portuguese (north/central) NBS programs. From the initiation of newborn screening by MS/MS in each region (in Galicia in 2000, in Andalusia in 2009, and in Portugal in 2004) until December 2011, the total number of MCADD cases detected were, 13 in Galicia (incidence 1:18736), 8 in Andalusia (incidence 1:23656) and 28 in north/central Portugal (incidence 1:11799). Global frequency is 1:15575. Four cases lacking sufficient genetic or follow-up data were excluded. The final ethnic breakdown was 31 Gypsy and 14 Caucasian patients.

At diagnosis, the following parameters were evaluated: age at which analytical samples were taken for NBS, presence or absence of clinical symptoms, C0, medium chain acylcarnitines (C8, C6, C10, C10:1) and ratios of C8/C2 and C8/C10 on the NBS blotter. Diagnosis was confirmed by mutation analysis of the *ACADM* gene. Clinical course was subsequently monitored.

During follow-up patients received a normal diet, according to age and avoiding prolonged fasting and lipolysis. A daily intake of 1-2 g/kg of slow absorption carbohydrates was recommended from eight months of age. During any acute intercurrent illness the treatment protocol was: careful management with frequent administration of drinks containing an appropriate amount of glucose until the patient improved. In case of vomiting or clinical deterioration, an urgent hospital admission for intravenous glucose infusion was recommended. Biochemical follow-up included measurement of C0 in blood spot/plasma at each visit (average frequency of 3 months) and an annual determination of general biochemical parameters including transaminase levels. Few data exist on the consequences of low carnitine in MCADD; supplementation was prescribed if C0 fell below 12 μmol/L in blood spots or below 20 μmol/L in plasma. Supplementation was only stopped after 2 independent determinations showed free carnitine higher than μmol/L in blood spots or 20 μmol/L in plasma. Carnitine was reintroduced if it fell again below the control values.

Informed consent was obtained from the parents of all patients. The study was approved by the Ethics Committee of each Hospital. Institutional Review Board: Fundación Ramón Dominguez G 15796683. Address: Travesía da Choupana s/n. 15706 Santiago de Compostela, A Coruña. Spain.

### Analytical methods

#### NBS

Acylcarnitines in blood spots were studied by the standard method of butylation and analysis by MS/MS. MCADD was suspected in a newborn if the medium chain acylcarnitines and/or C8/C2 or C8/C10 ratios were higher than the 99.9th percentile of the specific medium acylcarnitine (C8) and/or the ratios C8/C2 or C8/C10 (C8 p99.9 = 0.52 μM; C8/C2 p99.9 = 0.02; C8/C10 p99.9 = 1.8).

### Molecular testing

DNA was isolated and sequenced by standard procedures for blood samples of all patients and their parents, except for two patients conceived by in vitro fertilization with oocyte donation (Patients 1 and 16, Additional file 1), and whose biological mothers were not available for analysis. Molecular analysis of *ACADM* was performed using standard procedures. Primers were designed to overlap the coding sequences and their flanking regions (sequences available on request). PCR products were purified by ExoSap (usb®) enzyme and sequenced using a Big Dye Terminator Cycler Sequencing Ready reaction kit and the manufacturer’s protocol (Applied Biosystems). The sequencing reactions were performed in an ABI 3130XL Genetic Analyser.

### Biochemical follow-up

C0 was evaluated in blood spots or plasma samples. When using blood spots it was measured by MS/MS, according to the method of Zytkovicz et al [27]. In plasma, C0 was measured by the classical enzymatic/spectrophotometric assay [40].

When appropriate, data were statistically analyzed using the Student’s t-test (with p < 0.05 taken to indicate significance).

## Results

During the study period we evaluated 45 patients with MCADD detected by NBS, with the following geographical distribution: 26 cases from Portugal, 13 from Galicia, and 6 from Andalusia. The average age of analytical sample collection during screening was 7 days (range: 0-35), with a median of 5 days and a mode of 4 days. In 82.2% cases samples were obtained during the first week of life (37/45). In the remaining 8 cases (17.8%) repeat samples were analyzed subsequently, due to invalidity of the first sample. At diagnosis, all patients were asymptomatic with increased levels of medium chain acylcarnitines.

As shown in Additional file 1, all but patient 39 exhibited a marked elevation of C8 (average 4.8 ± 4.1 μmol/L; CV < 0.52). On the fourth day of postnatal age, patient 39 had a C8 value between percentiles 99.5 (0.37 μM) and 99.9 (0.52 μM), but a higher than normal C8/C2 ratio (0.04). A repeat sample, taken at 23 days of age showed a slight increase in C8 (0.54 μM) and C8/C2 (0.05), justifying a genetic study. The C8/C2 ratio was highly increased in all patients, with an average value of 0.28 ± 0.21 (CV < 0.02). A similar tendency was observed with the C8/C10 ratio, with an average value of 9.8 ± 5.7 μmol/L (CV < 1.85), but in patients 35 and 39 this ratio was normal (C8/C10 = 1.6 and 1). On re-sampling this ratio increased slightly (C8/C10 = 1.81). Increased levels of C10 or C10:1 was seen in nineteen and thirty nine patients respectively.

A diagnosis of MCADD was confirmed by molecular testing in all patients; 34 patients were homozygous for the prevalent c.985A>G (p.Lys329Glu) mutation and 10 patients were compound heterozygous for the prevalent and another mutation. Only patient 45 did not carry the c.985A>G mutation in any of the alleles (Additional file 1). Three of the mutations detected, c.600G>T (p.Trp200Cys), c.245G>C (p.Trp82Ser) and c.542A>G (p.Asp181Gly), are novel. Among our screened cases the prevalence of the common mutation was 86% (78 out of 90 alleles).

Levels of C8 and ratios of C8/C10 and C8/C2 were significantly higher (p = 0.016), while C0 was significantly lower (p < 0.001) in patients who were homozygous for the prevalent c.985A>G mutation, compared to with those with other genotypes (Additional file 1).

After diagnosis, all patients received dietary recommendations, but supplementation with L-carnitine (20-60 mg/kg/day) was prescribed only to thirty-two patients, having a low C0.

The average follow-up period was 3 years and 7 months (range 2 months-10 years 7 months). Follow-up data were available in 39 out of 45 cases (28 homozygous for the c.985A>G mutation and 11 with other genotypes). Eighty-two percent of patients (32/39) were given carnitine supplementation. Carnitine was not administered to 6 patients as their levels were consistently within the control range. One patient did not receive supplementation because his parents declined treatment, despite fulfilling the biochemical indication as per our protocol. Following carnitine treatment, the average C0 levels were significantly lower in patients homozygous for the common mutation than in patients with other mutations (14 μmol/L vs 22 μmol/L, p < 0.001) (Additional file 1). Among the 28 cases homozygous for the c.985A>G mutation, 26 (92.8%) required L-carnitine supplementation to reach normal levels, while fewer patients with other genotypes needed supplementation (6/11; 54.5%). The average follow-up period for homozygous patients was 31 months while the average for other patients was 18 months. According to our protocol, carnitine supplementation could never be stopped in eleven homozygous patients (34.3%). Supplementation was stopped in 15 homozygous patients but, reintroduction was necessary in 11 patients. Among 11 patients with a genotype other than homozygosity for the common mutation, carnitine supplementation was only required in one of them continuously (9.1%).

Two patients of Gypsy ethnicity (patients 4 and 33; Additional file 1) died at 15 and 32 months of age, respectively. Despite frequent carnitine supplementation, patient 4 consistently showed low C0 levels (average 10 μM). This patient died in a hospital not participating in the study, due to a lower respiratory tract infection. Patient 33 frequently showed low C0 levels (average 11.5 μM), but supplementation of carnitine was not always complied with. This patient died after presenting with gastroenteritis, decreased consciousness, hypotonia and in cardiorespiratory arrest.

Another three (patients 13, 17 and 21, Additional file 1), also of Gypsy ethnicity, suffered metabolic crises with episodes of hypoglycaemia and vomiting, triggered by infection. The remaining patients never had a metabolic crisis, although one (patient 31, Additional file 1) was also diagnosed with maple syrup urine disease.

## Discussion

C8 levels and several other ratios are utilized in screening for MCADD. We analysed the C8, C8/C2 and C8/C10 in our study and found C8/C2 ratio to be more accurate than either C8 and C8/C10, as it was elevated in all patients. By comparison, C8 was elevated in 44/45 and C8/C10 in 43/45 patients. We therefore consider the calculation of C8/C2 ration very important in screening for MCADD. This was exemplified in patients 35 and 39 whose diagnosis could easily have been missed without referral to the C8/C2 ratio. Both these patients were compound heterozygous for the common c.985A>G mutation as well as for other reported pathogenic mutations (c.683C>A [41] and c.199T>C [3]), respectively. It is thought that these latter two mutations might confer a more attenuated biochemical phenotype [3,41]. Patient 39 presented a biochemical profile similar to MCAD carrier, as also described Hsu et al. [42], and never showed clinical signs. The percentage of cases homozygous for the common mutation (76%) detected in our cohort was higher than previously reported, ranging from 30% to 71% (47.4% [3]; 43% [4]; 53% [19]; 61% [21]; 37% [27]; 71% [30]; 40% [31]; 63% [32]; 36.4% [33]; 52% [34]; 30% [35]). Our results could have a bias due to the high proportion of patients of Gypsy ethnicity (31/45, 69%), in whom the common allele shows a high population frequency [43]; 22/26 (84.6%) of our patients from Portugal were homozygous for the common mutation, while the remaining 12 homozygous patients were from Spain. We have previously observed that the prevalence for the common mutation in patients from Galicia (north-west Spain) was also high, at 63% [24].

The higher plasma C8 levels and C8/C10 and C8/C2 ratios that we have found in patients homozygous for the c.985A>G mutation agree with previous reports [3,4,20,23,24,34,38,44]. We noted a statistically significant association between low C0 level at NBS and homozygosity. As shown in Additional file 1, patients homozygous for the common mutation tended to maintain lower levels of C0 and carnitine supplementation is more frequent necessary in order that the plasma carnitine levels remain within the normal range. Treatment for MCADD is based mainly on diet: avoidance of fasting and ensuring high carbohydrate intake during illness. L-carnitine treatment has been proposed in MCADD therapy [45], although there is as yet no good evidence to support this recommendation. A survey of thirty-one centers in Europe, North America, Asia and Australia showed L-carnitine supplementation in MCADD to be controversial: 36% of the centers routinely used oral carnitine whereas 32% used it only in cases of proven carnitine deficiency or intercurrent infection [46]. Bzduch et al [47] reported a homozygous patient who had suffered two Reye-like episodes and whose C0 decreased continuously from an acute crisis for a further 8-13 days but then returned to normal by the 25^th^ day after the episode.

Apart from the c.985A>G mutation, it is well documented that most of the *ACADM* mutations seen in MCADD patients who have been diagnosed as a result of NBS are associated with asymptomatic or moderate clinical forms [48] of the disease. While it is also known that patients with these less commonly seen *ACADM* genotypes are also at risk of metabolic decompensation during periods of illness or metabolic stress [7,43,49,50], in our cohort clinical symptoms were only manifest in 11% of patients (5/45), all of whom were homozygous for the c.985A>G mutation.

In conclusion, our study points to that the *ACADM* genotype most commonly seen in MCADD might be of particular relevance in refining a follow-up protocol, since plasma carnitine levels in patients homozygous for c.985A>G tend to be lower and supplementation is required to maintain carnitine within the normal range. Nevertheless, this associations needs to be further supported by future studies with larger patients cohorts. Our results suggest that dietary management should be complemented with close monitoring of C0 levels and carnitine should be supplemented when necessary, and point to that this might be of particularly importance in patients homozygous for the common mutation. By demonstrating an association between carnitine levels and homozygosity for the c.985A>G mutation, the current study also contributes to our understanding of the relationship between genotype/biochemical markers and phenotype in MCADD. To a large extent, however, MCAD deficiency remains unpredictable, indicating the need for further prospective studies.

## Abbreviations

C10: Decanoylcarnitine (); FAO: Fatty acid β-oxidation; C0: Free carnitine; MCADD: Medium-chain acyl-CoA dehydrogenase deficiency; NBS: Newborn screening; C8: Octanoylcarnitine; MS/MS: Tandem mass spectrometry.

## Competing interests

None of the authors have any conflict of interest to declare.

## Authors’ contributions

MLC, PSP and HR reviewed the literature and conceived the study. LD was involved in patient selection, monitoring and data collection at Coimbra Hospital. ELT was involved in patient selection, monitoring and data collection at S Joao-Porto Hospital. EM was involved in patient selection, monitoring and data collection at María Pía-Porto Hospital. HS was involved in patient selection, monitoring and data collection at Gaia Hospital. MAB and CDP were involved in patient selection, monitoring and data collection at Virgen del Rocío Hospital. DEC and JAC were involved in patient selection, monitoring and data collection at Santiago de Compostela Hospital. JGV and AR interpreted the results of mutation analyses and were involved in the statistical analysis. MLC, AR and JMF reviewed and edited the manuscript. All authors critically revised the manuscript and approved the final version.

## Supplementary Material

Additional file 1: Table S1 Summary of levels of acylcarnitines at diagnosis, mutations, carnitine free levels, treatment and evolution of MCADD patients. Click here for file
