# Supplementary material for: Newborn screening for medium-chain acyl-CoA dehydrogenase deficiency: regional experience and high incidence of carnitine deficiency
Source: Orphanet J Rare Dis. 2013 Jul 10;8:102. doi: 10.1186/1750-1172-8-102 (PMC3718718; doi:10.1186/1750-1172-8-102)
Supplement: Additional file 1: Table S1 — Summary of levels of acylcarnitines at diagnosis, mutations, carnitine free levels, treatment and evolution of MCADD patients. [file 1750-1172-8-102-S1.docx]

|  | *Diagnosis* | | | | | | | | *Follow-up* | | | | |  |  |  |
| --- | --- | --- | --- | --- | --- | --- | --- | --- | --- | --- | --- | --- | --- | --- | --- | --- |
| Patients | Days at  collection | C0  (CV>12  µM) | C8  (CV<0.52  µM) | C10  (CV<0.50 µM) | C10:1  (CV<0.33 µM) | C8/C10  (CV<1.8) | C8/C2  (CV<0.02) | Genotype* | C0 median (range) (µM) bs (*cursive.ps)* | Carnitine supplementaion  (duration) | Initial dose  (mg/kg/day) | Follow up | Present  status | Dosis  carnitina |  |  |
| 1 | 4 | 14 | **1.3** | 0.20 | 0.27 | **6.6** | **0.12** | c.985A>G/  c.985A>G | 9.9 (5.4-15) | Yes (6y) | 20 | 10y 3m | FS |  | 6 | FS |
| 2 | 7 | 16 | **1.2** | 0.17 | 0.20 | **7.3** | **0.11** | c.985A>G/  c.985A>G | n.d. | No | - | 10y 1m | FS |  | 6 | FS |
| 3 | 10 | **9** | **3.0** | 0.38 | **0.66** | **7.9** | **0.34** | c.985A>G/  c.985A>G | 8.8 (3.7-15) | Yes (7y ) | 20 | 9 y | FS |  | 5 | FS |
| 4 | 23 | 21 | **5.8** | **0.56** | **0.47** | **10** | **0.67** | c.985A>G/  c.985A>G | 10 (6.3-15) | Yes (6m) | 20 | 1y 3m | Exitus |  | 1 | Exitus |
| 5 | 6 | 18 | **1.9** | 0.17 | 0.22 | **11** | **0.09** | c.985A>G/  c.985A>G | n.d. | No | - | 7y 5m | FS |  | 3 | FS |
| 6 | 4 | 41 | **9.4** | **0.71** | **0.97** | **13** | **0.74** | c.985A>G/  c.985A>G | 11 (4.2-17) | Yes (1y 6m) | 20 | 3y 2m | FS |  | 1 | FS |
| 7 | 4 | 35 | **4.6** | 0.40 | **0.55** | **11** | **0.55** | c.985A>G/  c.985A>G | n.d. | No | - | 3y 2m | FS |  | 1 | FS |
| 8 | 5 | 33 | **7.6** | **0.64** | **0.62** | **12** | **0.33** | c.985A>G/  c.985A>G | *42 (19.71)ps* | Yes (continuous) | 30 | 7y 1m | FS |  |  |  |
| 9 | 5 | 12 | **3.0** | 0.42 | **1.4** | **7.3** | **0.18** | c.985A>G/  c.985A>G | *32 (13-49)ps* | Yes (continuous) | 60 | 6y 9m | FS |  |  |  |
| 10 | 6 | 25 | **5.7** | 0.46 | **1.3** | **12** | **0.40** | c.985A>G/  c.985A>G | n.d. | No | - | 6y 1m | FS |  |  |  |
| 11 | 6 | 25 | **6.5** | **0.74** | **0.89** | **8.8** | **0.76** | c.985A>G/  c.985A>G | *50 (18-87)ps* | Yes (continuous) | 50 | 5y 6m | FS |  |  |  |
| 12 | 5 | 17 | **1.9** | 0.24 | **0.47** | **8.0** | **0.19** | c.985A>G/  c.985A>G | *50 (19-83)ps* | Yes (continuous) | 50 | 5y 6m | FS |  |  |  |
| 13 | 6 | 12 | **4.3** | 0.42 | 0.30 | **10** | **0.20** | c.985A>G/  c.985A>G | 16 (4.0-23) | Yes (3y 6m) | 30 | 5y 6m | MD |  |  |  |
| 14 | 7 | 29 | **13** | **1.1** | **0.42** | **11** | **0.31** | c.985A>G/  c.985A>G | *62 (16-83)ps* | Yes (continuous) | 50 | 4y 8m | FS |  |  |  |
| 15 | 5 | 20 | **1.8** | 0.27 | **0.66** | **6.7** | **0.14** | c.985A>G/  c.985A>G | 22 (10-36) | Yes (2y) | 30 | 4y 1m | FS |  |  |  |
| 16 | 13 | 22 | **2.0** | 0.16 | **0.36** | **13** | **0.27** | c.985A>G/  c.985A>G | 17 (13-20.3) | No | - | 2y 11m | FS |  |  |  |
| 17 | 6 | 27 | **6.9** | **0.59** | **1.1** | **12** | **0.46** | c.985A>G/  c.985A>G | 15 (11-21) | Yes (4m) |  | 1y 10m | MD |  |  |  |
| 18 | 3 | 29 | **21** | **1.3** | **1.0** | **16** | **0.85** | c.985A>G/  c.985A>G | *27 (18-34)ps* | Yes (7m) | 25 | 1y 7m | FS |  |  |  |
| 19 | 2 | 31 | **4.2** | 0.37 | **0.36** | **12** | **0.17** | c.985A>G/  c.985A>G | 12 (9.5-15) | Yes (continuous) |  | 1y 6m | FS |  |  |  |
| 20 | 4 | 24 | **5.7** | **0.55** | **0.55** | **11** | **0.15** | c.985A>G/  c.985A>G | n.d. | No | - | 1y 5m | FS |  |  |  |
| 21 | 4 | 31 | **2.5** | 0.24 | **0.34** | **11** | **0.10** | c.985A>G/  c.985A>G | 15 (5.6-21) | Yes (1y) | 30 | 2y 1m | MD |  |  |  |
| 22 | 8 | 20 | **2.8** | 0.25 | **0.36** | **11** | **0.26** | c.985A>G/  c.985A>G | 15 (11-19) | Yes (4m) | 30 | 1y 1m | FS |  |  |  |
| 23 | 0 | 27 | **8.1** | **0.73** | **1.06** | **11** | **0.56** | c.985A>G/  c.985A>G | 16 (10-22) | Yes (1y) | 60 | 1y 1m | FS |  |  |  |
| 24 | 4 | 37 | **9.7** | **0.61** | **0.81** | **16** | **0.39** | c.985A>G/  c.985A>G | 20 (11-33) | Yes (1y) | 30 | 1y 4m | FS |  |  |  |
| 25 | 20 | 22 | **4.4** | 0.44 | **0.95** | **9.9** | **0.26** | c.985A>G/  c.985A>G | n.d. | No | - | 6m | FS |  |  |  |
| 26 | 4 | 26 | **6.9** | **0.54** | **0.73** | **13** | **0.29** | c.985A>G/  c.985A>G | 17 (14-20) | Yes (3m) | 30 | 1y | FS |  |  |  |
| 27 | 5 | 28 | **2.4** | 0.41 | **0.35** | **5.8** | **0.21** | c.985A>G/  c.985A>G | 18 (11-26) | Yes (continuous) | 30 | 4m | FS |  |  |  |
| 28 | 3 | 20 | **3.0** | 0.38 | **0.76** | **8.1** | **0.13** | c.985A>G/  c.985A>G | 12 (3.9-20) | Yes (3m) | 20 | 1y 1m | FS |  |  |  |
| 29 | 35 | 14 | **2.3** | 0.45 | **0.56** | **5.2** | **0.28** | c.985A>G/  c.985A>G | 13 (3.1-29) | Yes (continuous) | 20 | 11m | FS |  |  |  |
| 30 | 13 | **11** | **1.4** | 0.18 | **0.53** | **7.8** | **0.07** | c.985A>G/  c.985A>G | 10 (5.0-15) | No | - | 2y | FS |  |  |  |
| 31 | 18 | 25 | **6.5** | **0.74** | **0.89** | **8.7** | **0.15** | c.985A>G/  c.985A>G | 15 (11-31) | Yes (continuous) | 30 | 1y 9m | FS |  |  |  |
| 32 | 5 | 18 | **2.8** | 0.44 | **0.85** | **6.9** | **0.17** | c.985A>G/  c.985A>G | 14 (9.9-30) | Yes (continuous) | 50 | 2y 8m | FS |  |  |  |
| 33 | 7 | 29 | **13** | **1.1** | **0.42** | **11** | **0.37** | c.985A>G/  c.985A>G | 11.5 (9.4-16) | Yes (continuous) | 20 | 2y 8m | Exitus |  |  |  |
| 34 | 4 | 21 | **1.4** | 0.15 | 0.04 | **9.9** | **0.06** | c.985A>G/  c.985A>G | 7.8 (4.9-12) | Yes (2y 2m) | 40 | 3y | FS |  |  |  |
| 35 | 6 | 22 | **0.8** | **0.50** | **0.33** | 1.6 | **0.07** | c.985A>G/  c.199T>C | 19 (11-29) | Yes (3m) | 20 | 9y 10m | FS |  | 6 | FS |
| 36 | 6 | 34 | **1.7** | 0.22 | **0.61** | **7.5** | **0.22** | c.245G>C /  c.985A>G | 16 (11-21) | Yes (6m) | 20 | 10y 7m | FS |  | 6 | FS |
| 37 | 3 | 45 | **1.3** | **0.51** | **0.39** | **2.4** | **0.12** | c.985A>G /  c.1247T>C | 28 (26-30) | No | - | 1y 7m | FS |  | 0.6 | FS |
| 38 | 2 | 34 | **16** | **1.3** | **1.8** | **12** | **0.12** | c.600G>T/ c.985A>G | 16 (9.5-25) | Yes (3m) | 20 | 1y 1m | FS |  |  |  |
| 39 | 4 | 35 | 0.42 | 0.39 | 0.22 | 1.0 | **0.04** | c.683C>A/ c.985A>G | 23 (21-30) | No | - | 2y | FS |  |  |  |
| 40 | 5 | 39 | **4.9** | **0.53** | **0.94** | **9.4** | **0.29** | c.985A>G/ c.1189dupT | *51 (15-85)ps* | Yes (continuous) | 50 | 4y 4m | FS |  |  |  |
| 41 | 4 | 44 | **2.0** | **0.79** | **0.57** | **2.5** | **0.05** | c.653C>G/ c.985A>G | 25 (12- 43) | Yes (3y) | 30 | 4y 5m | FS |  |  |  |
| 42 | 5 | 20 | **4.6** | 0.39 | **0.64** | **12** | **0.23** | c.985A>G/  n.f. | 25 (3.0-55) | Yes (8m) | 35 | 3y 10m | FS |  |  |  |
| 43 | 6 | 56 | **3.1** | 0.29 | **0.41** | **11** | **0.12** | c.250C>T/ c.985A>G | 28 (18-46) | No | - | 2m | FS |  |  |  |
| 44 | 4 | 25 | **1.2** | 0.23 | **0.33** | **5.2** | **0.07** | c.250C>T / c.985A>G | 20 (15-23) | No | - | 2y 10m | FS |  |  |  |
| 45 | 4 | 43 | **5.2** | **0.81** | **0.56** | **6.4** | **0.60** | c.542A>G/ c.799G>A | 20 (14-23) | No | - | 1y 10m | FS |  | 0.8 | FS |

Table 1. Summary of levels of acylcarnitines at diagnosis, mutations, carnitine free levels, treatment and evolution of MCADD patients.

CV: control values; FS: Free of Symptoms; MD: Metabolic decompensation; n.d.= not done; n.f: not found in this moment m: month; y: year; bs (blood spot samples); *ps (plasma samples)*

*All mutations were named according to the nucleotide change.
